# Central line-associated *Cyberlindnera fabianii* fungemia: A case report and review of diagnostic and therapeutic challenges

**DOI:** 10.5339/qmj.2025.90

**Published:** 2025-09-16

**Authors:** Sreethish Sasi, Gawahir A. Ali, Husam Salah, Wael Goravey, Muna Al Maslamani

**Affiliations:** 1 Infectious Diseases Division, Department of Medicine, Communicable Diseases Center, Hamad Medical Corporation, Doha, Qatar; 2 Microbiology Division, Department of Laboratory Medicine and Pathology, Hamad Medical Corporation, Doha, Qatar; 3 College of Medicine, Qatar University, Doha, Qatar *Email: ssasi7@hamad.qa

**Keywords:** *Cyberlindnera fabianii*, fungemia, central venous catheter, echinocandin therapy, MALDI-TOF, case report, State of Qatar

## Abstract

**Introduction:**

*Cyberlindnera fabianii* is an uncommon opportunistic yeast increasingly recognized as a cause of invasive fungal infections, particularly in immunocompromised patients and those with indwelling medical devices. Clinical experience remains limited, with most published cases involving neonates or adults with significant comorbidities.

**Case Presentation:**

We report a case of *C*. *fabianii* fungemia in a 26-year-old man with end-stage renal disease on maintenance hemodialysis via a long-term tunneled catheter. The patient presented with fever following dialysis and was found to have leukocytosis and elevated inflammatory markers. Blood cultures from both peripheral and catheter sites grew yeast after 48 hours. Empiric antibacterial therapy was initiated, and the hemodialysis catheter was removed. Antifungal treatment with anidulafungin was started, leading to clinical improvement. Species identification was achieved using matrix-assisted laser desorption/ionization time-of-flight mass spectrometry (MALDI-TOF MS), which revealed *C*. *fabianii*. Antifungal susceptibility testing demonstrated low minimum inhibitory concentrations (MICs) for echinocandins and variable susceptibility to azoles. The patient completed a 14-day course of anidulafungin with full recovery.

**Discussion:**

This case underscores the importance of accurate identification of rare yeasts such as *C*. *fabianii*, which may be misidentified as other less pathogenic species. MALDI-TOF MS and molecular diagnostics are critical tools for early detection. Due to its potential for azole resistance and biofilm formation, echinocandins appear to be an effective treatment option. Prompt catheter removal and appropriate antifungal therapy were pivotal to the patient’s successful outcome.

**Conclusion:**

*C*. *fabianii* should be considered in patients with fungemia and risk factors for invasive candidiasis, especially when initial identification is inconclusive. Awareness of this emerging pathogen and its management is essential to ensure timely intervention and improve clinical outcomes.

## 1. INTRODUCTION

*Cyberlindnera fabianii* (formerly known as Hansenula fabianii, Pichia fabianii, and Lindnera fabianii) is an ascomycetous yeast that is a rare cause of human infection.^1^ Since its first description in 1948, only a few cases of invasive *C*. *fabianii* infection have been reported in the literature.^1^ Although this organism is generally considered to have low virulence, it has been implicated in serious infections in high-risk patients. Published case reports have described *C*. *fabianii* as a cause of endocarditis,^2^ pneumonia, catheter-related bloodstream infections, prostatitis, central nervous system infections, and other invasive diseases, particularly in immunocompromised hosts.^1,3–7^ Most reported patients have well-recognized predisposing factors similar to those for invasive candidiasis—notably the presence of central venous catheters (CVCs), recent broad-spectrum antibiotic use, prematurity, malignancy, surgery, or immunosuppression.^1,3–8^ For example, a neonatal intensive care unit outbreak in Kuwait in 2019 involved 10 preterm infants with *C*. *fabianii* fungemia, all of whom had received prior antibiotics and had indwelling catheters. Two of those neonates died because of sepsis and multiorgan failure, despite treatment, underscoring the emerging pathogenic potential of this unusual yeast.^6^ Fatal cases have also been documented in immunocompromised adults, including a leukemia patient who developed *C*. *fabianii* fungemia after cord blood transplant, and succumbed to it due to multi-organ failure.^4^ Accurate identification of *C*. *fabianii* remains challenging. Commercial biochemical identification systems frequently misidentify this yeast as Candida utilis (synonym Candida jadinii) or as Wickerhamomyces anomalus, which may not only lead to underestimation of its clinical incidence but also impact treatment decisions and patient outcomes. These misidentified species are often presumed susceptible to fluconazole, potentially delaying the initiation of more effective agents like echinocandins. Such delays can result in persistent fungemia and increased risk of complications, especially in vulnerable patients with indwelling catheters or immunosuppression.^8^ In contrast, modern molecular and proteomic methods improve diagnostic accuracy. Matrix-assisted laser desorption/ionization time-of-flight mass spectrometry (MALDI-TOF MS) can often identify *C*. *fabianii* to the species level,^8,9^ though rare isolates may still require sequencing of ribosomal DNA for definitive identification.^5^ This organism’s ability to form biofilms is thought to contribute to its antifungal resistance profile.^1^ Prior studies have noted *C*. *fabianii* ’s propensity for developing resistance to multiple antifungal classes, including azoles (fluconazole, voriconazole), amphotericin B, and even echinocandins in certain cases.^8^ Notably, fluconazole prophylaxis has failed in about 50% of *C*. *fabianii* infections, with progression to fatal septicemia reported.^8^ There are no established treatment guidelines due to the rarity of this pathogen; management is typically guided by clinical experience and general Candida infection guidelines.^1^ Here we report a case of central line-associated *C*. *fabianii* fungemia in a hemodialysis patient, highlighting the diagnostic challenges, resistance patterns, and successful treatment with an echinocandin in the context of the existing literature. The patient was treated at Hamad Medical Corporation, the largest public sector healthcare provider in Qatar.^10^ The manuscript of this case report was approved by the Institutional Review Board, Medical Research Centre (MRC) of Hamad Medical Corporation (MRC-04-23-732) on 27/10/2023. Written informed consent was obtained from the patient for publication.

## 2. CASE PRESENTATION

A 26-year-old man with end-stage renal disease (ESRD) on maintenance hemodialysis via a right internal jugular tunneled catheter (permcath, in place for 3 months) was brought to our emergency department (ED) by the ambulance service, with one day of fever and malaise following an otherwise routine dialysis session. He had no other symptoms such as cough, dyspnea, or abdominal pain. On examination, he was febrile (38.4°C) and tachycardic (108 beats/min) with blood pressure of 125/68 mmHg and oxygen saturation of 97% on room air. The hemodialysis catheter site appeared clean with no erythema or tenderness, and no other obvious source of infection was identified. Laboratory investigations were notable for leukocytosis (white blood cell count, 20.3×10^9^/L, normal 4–11) and elevated inflammatory markers (C-reactive protein = 251 mg/L [normal <5], procalcitonin 6 µg/L [normal <0.05]). Chest radiography was unremarkable. Empiric broad-spectrum antibacterial therapy with piperacillin-tazobactam was initiated for possible sepsis while awaiting culture results. The patient was then admitted under the internal medicine department with consultations to infectious diseases and nephrology.

**Figure 1:**
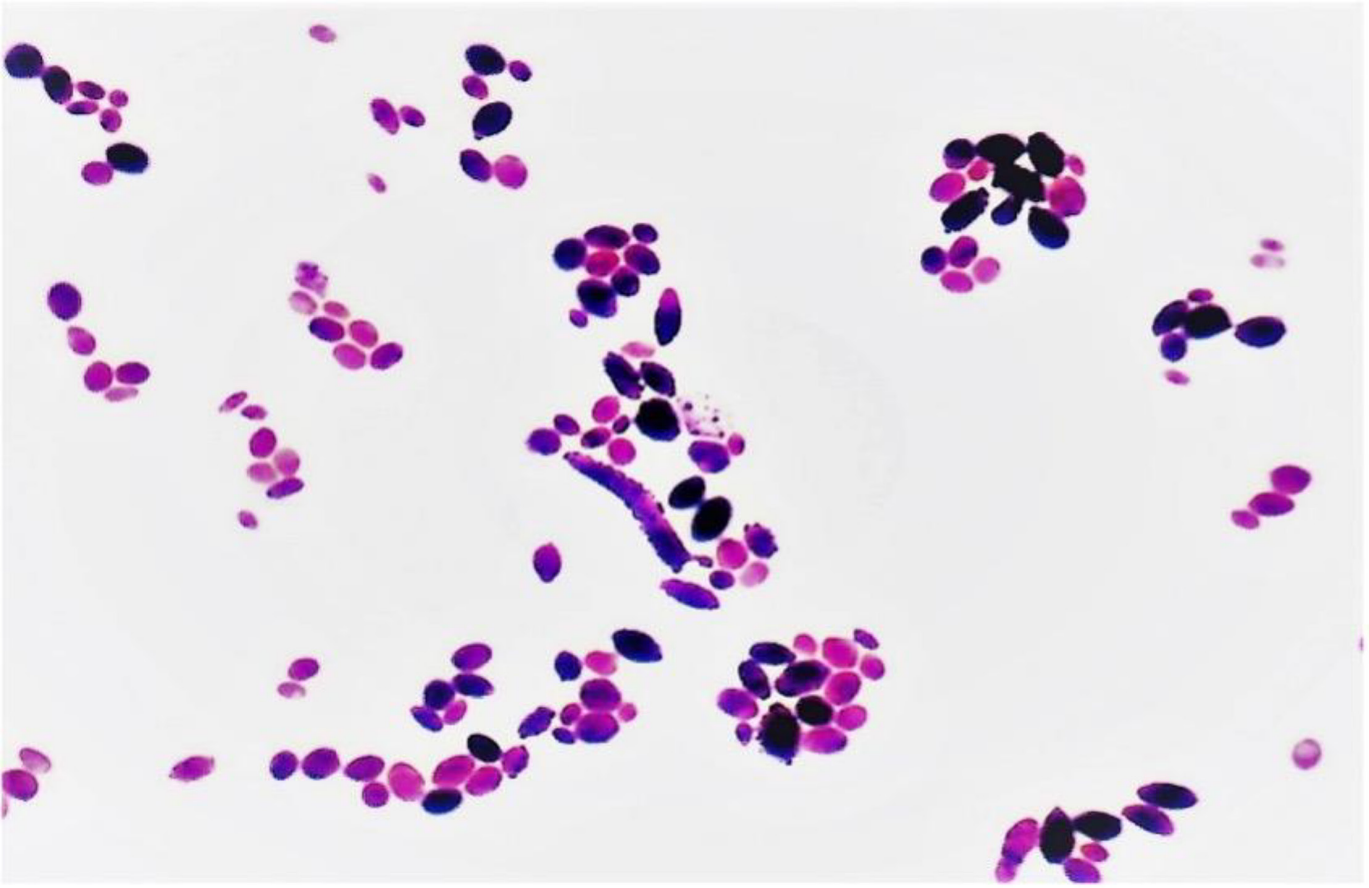
Gram stain of *Cyberlindnera fabianii* from positive blood culture. # Gram stain (original magnification 1000×, oil immersion) reveals oval to ellipsoid budding yeast cells consistent with *C. fabianii*. The morphology initially suggested a *Candida species* prior to MALDI-TOF confirmation.

**Figure 2:**
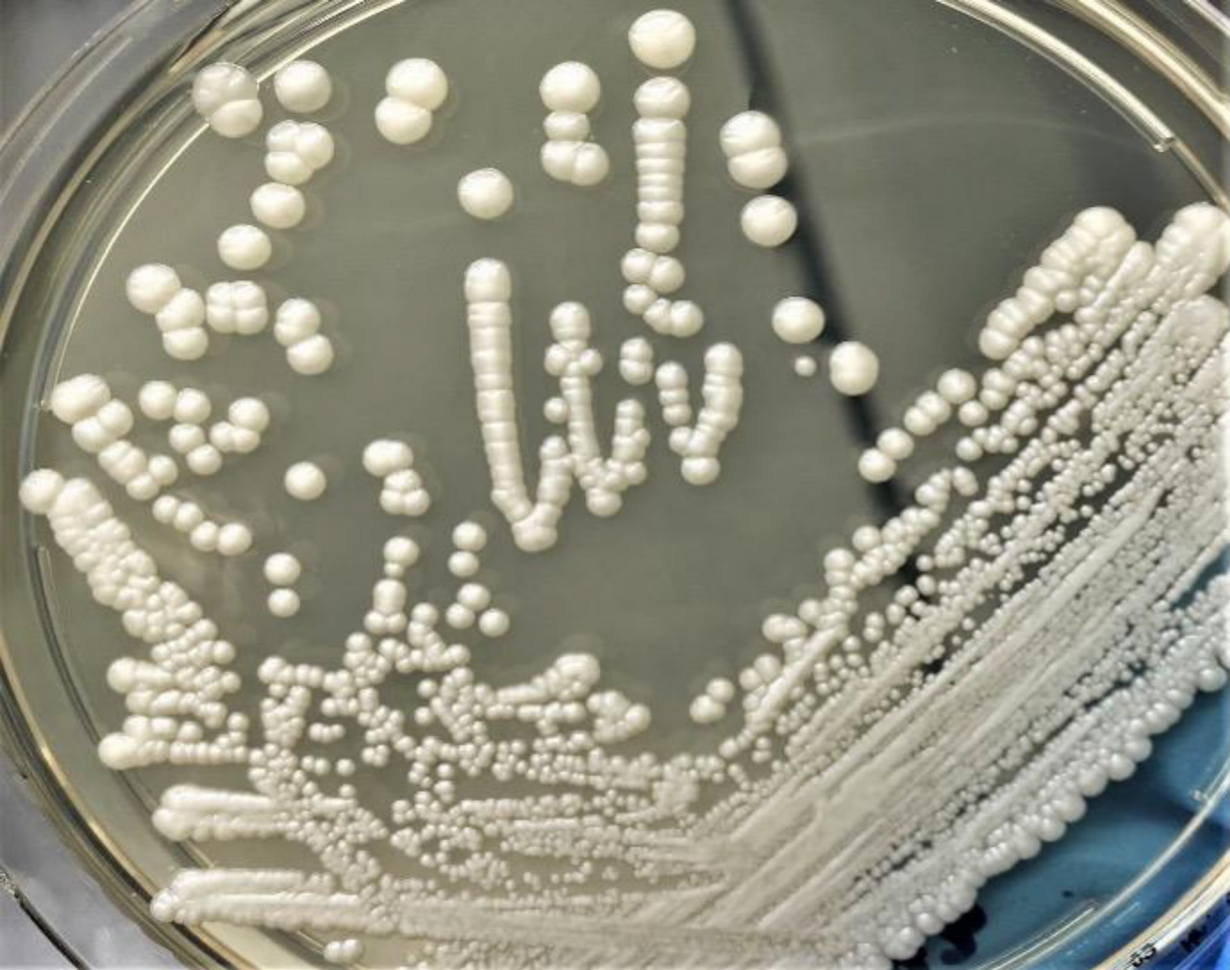
Colonial morphology of *Cyberlindnera fabianii* on Sabouraud dextrose agar. # Creamy white yeast colonies appeared after 48 hours of incubation at 35°C. The culture was derived from blood specimens taken from both the peripheral vein and the central venous catheter.

After 48 hours of incubation, both peripheral blood and catheter-drawn blood cultures signaled positive, yielding oval to ellipsoid budding yeast cells on Gram stain (Figure 1). Creamy white yeast colonies grew on Sabouraud dextrose agar (Figure 2). In light of the presumptive candidemia, the hemodialysis catheter was promptly removed, and antifungal therapy was started empirically with anidulafungin (200 mg intravenous loading dose, then 100 mg IV daily). Starting empirical antifungal therapy with anidulafungin (or caspofungin) in the context of presumptive candidemia, particularly catheter-related in high-risk patients, is our routine clinical practice. Following removal of the infected right internal jugular tunneled catheter, the patient underwent hemodialysis via a temporary femoral catheter. A new tunneled dialysis catheter was placed in the contralateral (left) internal jugular vein after clinical stabilization and documentation of sterile blood cultures at 72 hours post-antifungal initiation.

Identification of the yeast was performed by MALDI-TOF MS (Bruker Biotyper System), which provided a confident identification of *C*. *fabianii* (score 2.34). Subsequent antifungal susceptibility testing of the isolate was done using the Sensititre YeastONE YO10 panel. The minimum inhibitory concentrations (MICs) indicated that the organism was susceptible to echinocandins (MIC for anidulafungin 0.03 μg/mL; caspofungin 0.03 μg/mL; micafungin 0.03 μg/mL) and azoles such as voriconazole (0.03 μg/mL) and itraconazole (0.25 μg/mL), with reduced susceptibility to fluconazole (MIC 2 μg/mL) and intermediate susceptibility to amphotericin B (MIC 1 μg/mL; Table 1). These results confirmed that our initial choice of an echinocandin was appropriate.

The patient’s clinical condition improved rapidly after catheter removal and initiation of antifungal therapy. Follow-up blood cultures obtained 72 hours after starting anidulafungin remained sterile. A transthoracic echocardiogram revealed no evidence of endocardial vegetations, and ophthalmologic examination showed no signs of endophthalmitis. A transthoracic echocardiogram was performed to rule out infective endocarditis, as *C*. *fabianii* has been rarely reported to cause this complication. Ophthalmologic examination was done to exclude fungal endophthalmitis, a known risk in fungemia, although specific ocular involvement by *C*. *fabianii* is not well documented. The patient completed a two-week course of IV anidulafungin. He was discharged home on day 15 after admission, after completing his antifungal therapy. He defervesced within a few days of therapy and had an uneventful recovery. At outpatient follow-up one month after hospital discharge, he was clinically well with no recurrence of fungemia. A follow-up visit approximately 2 to 4 weeks after hospital discharge is a common practice for patients treated for candidemia or other fungemias, including those caused by uncommon yeasts like *C*. *fabianii*.

## 3. DISCUSSION 

*C*. *fabianii* is an uncommon opportunistic yeast, and clinical experience with this pathogen is limited to case reports and small series. A 2019 review of published cases identified 39 cases of *C*. *fabianii* infection reported between 1990 and 2018, the vast majority (≈90%) of which were documented in the last decade.^8^ This suggests increasing recognition of *C*. *fabianii* as an emerging pathogen. The infection has a predilection for two patient populations: neonates and individuals with significant comorbidities.^3,4,7^ Nearly half of the reported cases have been in neonates (often premature infants), while the remainder occurred in older children and adults.^8^ Across these cases, certain risk factors are consistently observed. *C*. *fabianii* infections typically occur in hosts with recent antibiotic exposure, indwelling CVCs, or other risk factors for invasive candidiasis.^4,7,8^ Low birth weight, intensive care unit stays, malignancy, neutropenia, renal failure, and recent surgery have all been noted as contributing factors.^6,8^ Our patient had multiple risk factors—renal failure, an indwelling hemodialysis catheter, and recent broad-spectrum antibiotic therapy—which align with those reported in the literature and likely facilitated this unusual fungemia. Indeed, central lines are frequently implicated; most cases of *C*. *fabianii* fungemia have been catheter-related, and removal of the catheter is regarded as a crucial step in management. In our case, immediate removal of the infected catheter was undertaken, which is consistent with current recommendations for any catheter-associated fungal bloodstream infection.^11,13^

Another significant concern in *C*. *fabianii* infections is antifungal resistance. *C*. *fabianii* is known to form biofilms on medical devices, and this attribute may contribute to its reduced susceptibility to antifungal agents, particularly azoles.^1^ Biofilm-associated organisms are shielded from drug penetration, and in *C*. *fabianii*, this has been linked to cross-resistance among azoles.^1^ Hamal et al reported a case of *C*. *fabianii* (as Pichia fabianii) aortic valve endocarditis in which prolonged therapy with fluconazole followed by voriconazole led to the emergence of strains with markedly elevated MICs to both agents.^2^ The development of multi-azole resistance in that patient was attributed to biofilm formation and adaptive resistance, as the yeast was able to evade the antifungal effects by altering its phenotype.^1,2,8^ In addition to azoles, there are reports of *C*. *fabianii* developing resistance to amphotericin B and even to caspofungin in vitro, although clinical data on echinocandin resistance are scarce. Overall, susceptibility data for *C*. *fabianii* remain limited given the small number of isolates studied, but what is available indicates considerable inter-strain variability.^2,12^ For instance, Al-Sweih et al noted all outbreak isolates had reduced susceptibility to triazoles,^6^ and in a 2019 analysis^8^ of six clinical isolates, fluconazole MICs ranged from 1 to 8 μg/mL—significantly higher than those of other antifungals. In the psoas abscess case from 2023, the *C*. *fabianii* isolate demonstrated a fluconazole MIC of 32 μg/mL, by far the highest among the tested drugs.^1^ In contrast, echinocandin and voriconazole MICs in that case were very low (≤0.06 μg/mL).^1^ Consistently, studies have found that echinocandins are the most active class against *C*. *fabianii*, often with MIC values in the 0.006 to 0.03 μg/mL range.^8^ This generally favorable echinocandin susceptibility, even in isolates with azole resistance, is an important feature that influences treatment decisions.

Given the above, management of *C*. *fabianii* fungemia presents several challenges. There are no species-specific guidelines, so clinicians must extrapolate from guidelines for invasive candidiasis and from reported case outcomes. The Infectious Diseases Society of America (IDSA) candidiasis guidelines recommend an echinocandin as first-line therapy for candidemia in patients with recent azole exposure, severe illness, or when non-albicans Candida species are suspected.^13^ *C*. *fabianii* infections often fall into this category of “non-albicans” candidemia, where an echinocandin is preferred empirically, especially given the frequent azole resistance. In our patient, although there was no prior azole prophylaxis, the decision to start an echinocandin (anidulafungin) empirically was prudent in the context of suspected catheter-related candidemia. This approach is supported by the literature—for example, Lee et al successfully treated a *C*. *fabianii* bloodstream infection with 14 days of anidulafungin, resulting in full recovery.^3^ Likewise, our patient responded well to a two-week anidulafungin course with catheter removal. Echinocandins have also been used as step-down or combination therapy in neonates and other cases, although amphotericin B has been the mainstay in some neonatal infections.^6^ In the Kuwaiti neonate outbreak, most infants were treated with amphotericin B (often combined with flucytosine or followed by fluconazole), and 8 of 10 survived.^6^ This suggests amphotericin B is effective in many cases, but its use may be limited by patient tolerance (especially in adults with comorbidities) and by the availability of echinocandins as safer alternatives. Removal of any infected device (catheters, shunts, etc.) is uniformly recommended, as persistent fungemia is unlikely to clear with antifungals alone if a biofilm-laden device remains in place.^11,13^ In our case, the rapid clearance of fungemia after catheter removal and appropriate therapy highlights the importance of this intervention. There is no universally fixed interval for routine replacement of long-term hemodialysis catheters (e.g., tunneled CVCs like permcaths) in asymptomatic patients. Instead, current guidelines and literature emphasize retaining the catheter as long as it remains functional and free of complications.^14,15^

Outcomes in reported *C*. *fabianii* infections have been variable, largely depending on the host’s condition and the timeliness of appropriate therapy. Many adult patients and older children have had favorable outcomes when the infection is recognized early and treated with an effective antifungal (often an echinocandin or amphotericin) in conjunction with source control. For instance, recent case reports of catheter-related *C*. *fabianii* fungemia (including this case and others) describe cure with no recurrence.^3,7^ On the other hand, mortality has been observed in the most fragile patients. Two neonates in the aforementioned outbreak died despite treatment, one of them before antifungal therapy could be optimized.^6^ Another reported neonate case—a premature infant with complex cardiac disease – succumbed to septic shock from *C*. *fabianii* peritonitis despite receiving antifungal treatment, likely due to the infant’s severe underlying condition and the invasive nature of the infection.^5^ These cases illustrate that *C*. *fabianii* fungemia can lead to poor outcomes if not promptly and aggressively managed. Early diagnosis and targeted therapy are therefore critical. As noted by Lee et al, timely identification of this unusual yeast and initiation of effective therapy are essential to improve prognosis and avoid potentially devastating complications.^3^ Our experience reinforces this point: swift microbiological diagnosis (via MALDI-TOF) and early use of an appropriate antifungal (anidulafungin) were key to the successful outcome.

## 4. CONCLUSION

*C*. *fabianii* fungemia is an emerging opportunistic infection associated with indwelling central lines and other risk factors commonly seen in invasive candidiasis. In this report, we describe a young adult with ESRD who developed *C*. *fabianii* fungemia related to a tunneled hemodialysis catheter. Prompt catheter removal, early initiation of anidulafungin, and accurate species identification led to complete clinical recovery without recurrence. This case highlights the importance of considering rare yeasts like *C*. *fabianii* in patients with candidemia who fail to grow typical Candida species, especially in those with prior antibiotic exposure or long-term catheters. Accurate identification using MALDI-TOF or molecular methods is crucial, as routine methods may misidentify this pathogen and potentially lead to suboptimal therapy. *C*. *fabianii* often exhibits reduced susceptibility to fluconazole and can develop resistance during azole treatment, whereas echinocandins and amphotericin B generally retain good activity. Removal of the infection source (such as CVCs) and prompt initiation of effective antifungal therapy are pivotal for a favorable outcome. In our patient, these interventions led to a complete cure. As reports of *C*. *fabianii* infections accumulate, increased awareness and early intervention will improve the management of this rare but potentially serious fungemia. Our case adds to the growing literature and underscores the need for continued vigilance and research regarding this unusual fungal pathogen.

## LIST OF ABBREVIATIONS

**Table:** 

CVC	Central venous catheter
ESRD	End-stage renal disease
IDSA	Infectious Diseases Society of America
MALDI-TOF MS	Matrix-Assisted Laser Desorption/Ionization Time-of-Flight Mass Spectrometry
MIC	Minimum inhibitory concentration

## DATA AVAILABILITY

Data supporting the conclusions of the study are all available free of cost through open-access journals and websites.

## STATEMENT OF ETHICS

The manuscript of this case report was approved by the Institutional Review Board (IRB), Medical Research Centre (MRC) of Hamad Medical Corporation (MRC-04-23-732) on 27/10/2023.

## AUTHOR CONTRIBUTIONS

Patient Management & Clinical Data Acquisition: GAA, WG. Microbiological Analysis & Laboratory Investigations: HS. Case Analysis & Interpretation: SS, GAA, WG. Manuscript Drafting & Literature Review: SS, GAA, WG. Critical Revision of Manuscript: HS, MAM. Supervision & Final Approval: HS, MAM.

## FUNDING INFORMATION

The research reported in this publication was supported by the Medical Research Center (MRC) of Hamad Medical Corporation (HMC). The research reported in this publication was supported by Qatar National Library under the QNL open access program.

## ACKNOWLEDGEMENTS AND DISCLOSURES

The authors acknowledge the support of the Infectious Diseases and Microbiology Divisions of Hamad Medical Corporation and the Medical Research Center (MRC) of Hamad Medical Corporation.

## PATIENT CONSENT

Written and informed consents were obtained from the patients for the publication of their case information and images.

## CONFLICTS OF INTEREST

The authors have no conflicts of interest to declare.

## Figures and Tables

**Table 1.** Minimum inhibitory concentrations (MICs) of antifungal agents for *Cyberlindnera fabianii* isolate.

Antifungals	MIC (µg/mL)
Flucytosine	0.25
Fluconazole	2
Itraconazole	0.25
Posaconazole	0.5
Voriconazole	0.03
Micafungin	0.03
Anidulafungin	0.03
Caspofungin	0.03
Amphotericin B	1
